# Effects of TiO_2_ structure and Co addition as a second metal on Ru-based catalysts supported on TiO_2_ for selective hydrogenation of furfural to FA

**DOI:** 10.1038/s41598-021-89082-x

**Published:** 2021-05-07

**Authors:** Weerachon Tolek, Natdanai Nanthasanti, Boontida Pongthawornsakun, Piyasan Praserthdam, Joongjai Panpranot

**Affiliations:** Center of Excellence on Catalysis and Catalytic Reaction Engineering, Department of Chemical Engineering, Faculty of Engineering, Chulalongkorn University, Bangkok, 10330 Thailand

**Keywords:** Heterogeneous catalysis, Chemical engineering

## Abstract

The TiO_2_ supported Ru-based catalysts were prepared with 1.5 wt% Ru and 0–0.8 wt% Co on various TiO_2_ (anatase, rutile, P-25, and sol–gel TiO_2_) and studied in the liquid-phase selective hydrogenation of furfural to furfuryl alcohol (FA) under mild conditions (50 °C and 2 MPa H_2_). The presence of high anatase crystallographic composition on TiO_2_ support was favorable for enhancing hydrogenation activity, while the strong interaction between Ru and TiO_2_ (Ru–TiO_x_ sites) was required for promoting the selectivity to FA. The catalytic performances of bimetallic Ru–Co catalysts were improved with increasing Co loading due to the synergistic effect of Ru–Co alloying system together with the strong interaction between Ru and Co as revealed by XPS, H_2_-TPR, and TEM–EDX results. The enhancement of reducibility of Co oxides in the bimetallic Ru–Co catalysts led to higher hydrogenation activity with the Ru–0.6Co/TiO_2_ catalyst exhibited the best performances in FA selective hydrogenation of furfural to FA under the reaction conditions used.

## Introduction

Due to an increasing global demand of energy and chemicals in addition to the concerning about the environmental impact and the decline of petroleum reserves, the non-fossil carbon resources have gained much attention as the alternative renewable resource. Lignocellulose biomass have emerged as the most abundant, potential, and economical resources for the alternative and sustainable feedstock to produce biofuel and biochemical. Especially the production of biochemical instead of fine chemical-derived petroleum is more interesting alternative than that of biofuel production in order to increase the economic value. Among biomass-derived chemicals, furfural obtained from lignocellulose biomass is considered as one of the key intermediate platforms for the synthesis of various chemicals and fuels. The furfural-derived biomass can be produced by acid-catalyzed dehydration of xylose or by fast pyrolysis of biomass^1^.

According to furfural applications, it has been reported that 62% of furfural produced is used for the synthesis of furfuryl alcohol (FA)^2^, which is the important chemical in various applications including polymer, resin, synthetic fiber, vitamin C, lysine, lubricant, agrochemical, and perfume^2–5^. FA is synthesized via selective hydrogenation of aldehyde C=O functional group to alcohol O–H group. In industry, the selective hydrogenation of furfural to FA could be carried out in vapor or liquid phase^6,7^. Most commonly used method for FA synthesis is proceeded on copper chromite catalysts at high pressure and elevated temperature for decades^8,9^. The performances of copper chromite catalysts have been reported to show moderate activity in furfural conversion^5,8,10^. Furthermore, toxicity due to the chromium oxides is mainly considered as the great disadvantage in terms of environmental pollutants^11^. Thus, this has led to research interest for the development of Cr-free catalysts, which are environmentally friendly catalysts for FA synthesis with high activity and selectivity.

Group VIII metals based catalysts (i.e., Ru, Rh, Pd, Os, Ir, and Pt) are expected to become the promising catalysts for hydrogenation of furfural as an alternative to the conventional copper chromite catalysts because of their ability to reduce the carbonyl C=O group in the hydrogenation reactions^8^. Among them, ruthenium (Ru) seems to be interesting for the hydrogenation of functional C=O group and is a challenge to modify the catalytic performances. For example, the Ru/ZnO catalyst was reported to be the selective catalyst for the selective hydrogenation of crotonaldehyde to crotyl alcohol (selectivity up to 88%)^12^. For the catalytic transfer hydrogenation of furfural, the monometallic 5 wt% Ru/C showed moderate furfural conversion (68%) but low selectivity to FA (12%) under 180 °C and 2.04 MPa N_2_ for 5 h^13^. However, the performances of Ru on different carbon supports have been reported that the furfural conversion of 1.5 wt% Ru/carbon nanotubes was very low (14%) but selectivity to FA was high up to 88% at temperature of 90 °C and 2 MPa H_2_, while the 1.5 wt% Ru/carbon black still showed similar performances (23% furfural conversion and 63% selectivity to FA) under mild conditions (50 °C and 0.5 MPa H_2_)^11^. In addition, the 2.6 wt% Ru/Zr-MOFs was found to be efficient catalyst (94.9% yield of FA) for selective hydrogenation of furfural to FA under mild conditions (20 °C and 0.5 MPa H_2_ for 4 h)^14^. Thus, it could suggest that the support played as one of crucial roles on the performances of furfural hydrogenation to FA.

Titanium dioxide (titania: TiO_2_) has been widely used as a support for active catalysts because of its ability to modify the catalytic properties of the supported phase. The structure of titania support was found to significantly affect the catalytic performances of catalysts in hydrogenation reaction because different titania structures would result in different physio-chemical properties and catalytic properties such as dispersion, particle size, and reduction behavior of active phase. For example, the anatase TiO_2_ supported Ni catalyst showed higher catalytic activity in *p*-nitrophenol hydrogenation as compared to rutile TiO_2_ supported Ni catalyst due to the ease of reduction behavior from Ni oxides to Ni^0^ metal over the anatase TiO_2_ support^15^. Owing to various structures of TiO_2_, the TiO_2_ support is a promising support to modify the performances of Ru-based catalysts for hydrogenation of furfural to FA.

Moreover, the modification of Ru-based catalysts for furfural hydrogenation has been attempted by adding a second metal. The tin addition onto Ru/C catalyst with Sn/Ru ratio of 0.4 could promote the hydrogenation of furfural in terms of conversion and selectivity to FA (90 °C and 1.25 MPa) due to the dilution effect and the close interaction between Ru and Sn^8^. Not only hydrogenation of furfural, but also the hydrogenation of C=O bond of crotonaldehyde to crotyl alcohol and other unsaturated fatty acids and their esters to their unsaturated alcohols have been reported over the bimetallic RuSn catalysts^16–18^.

Concerning the modification by the second metal addition, many research attempts to improve the catalytic performances of the catalyst for the hydrogenation of functional C=O group have shown the modification by Co addition^19–22^. Furthermore, the Co addition as a second metal has also been reported in the modification of Cu-based catalysts for selective hydrogenation of furfural to FA and it was found that the resulting bimetallic CuCo/carbon matrix catalyst with Co/Cu molar ratio of 0.4 was efficient in the furfural hydrogenation in terms of conversion, selectivity, and stability because of small particle size, high dispersion and synergistic effect of Cu and Co^10^. The modification of Ru-based catalysts by Co addition, however, has not been studied to much of a degree in the catalytic hydrogenation of functional C=O group, especially furfural.

This paper aims to study the liquid-phase selective hydrogenation of furfural to FA under mild conditions (50 °C and 2 MPa H_2_) by using the Ru-based catalysts supported on different TiO_2_ structures. Based on the Ru catalysts supported on suitable TiO_2_ structure, the modification by Co addition as a second metal was also investigated. Their catalyst properties and characteristics were investigated by means of X-ray diffraction (XRD), N_2_ physisorption, CO pulse chemisorption, H_2_-temperature-programmed reduction (H_2_-TPR), X-ray photoelectron spectroscopy (XPS), and transmission electron spectroscopy-energy dispersive X-ray spectroscopy (TEM–EDX).

## Experimental

### Preparation of TiO2 support

The TiO_2_ supports used for the preparation of Ru-based catalysts in this work were consisted of TiO_2_ anatase (Alfa Aesar), TiO_2_ rutile (Sigma Aldrich), TiO_2_-P25 (Degussa), and TiO_2_ prepared by sol–gel method. The TiO_2_ mixed phase support was prepared by using sol–gel method as follows. Approximately 83.5 cm^3^ of titanium isopropoxide (Sigma Aldrich) as TiO_2_ precursor was added into the solution containing nitric acid (HNO_3_, 65 vol%) in deionized water (7.3 cm^3^ HNO_3_: 1000 cm^3^ H_2_O). Then, the mixture solution was continually stirred at room temperature for 3 day until the clear sol was obtained. The clear sol was placed and dialyzed in cellulose membrane, which was submerged in the deionized water, for 3 day. During dialyzing, the water was daily changed until the pH of water reached about 3.5. After that, the resulting sol was dried in oven at 110 °C for 12 h. The dried sol was milled and then calcined in air flow at 350 °C for 2 h with heating rate of 10 °C/min.

### Preparation of the monometallic Ru/TiO_2_ and Co/TiO_2_ catalyst

The monometallic 1.5 wt% Ru catalysts supported on different TiO_2_ supports, consisted of TiO_2_ anatase (Alfa Aesar), TiO_2_ rutile (Sigma Aldrich), TiO_2_-P25 (Degussa), and TiO_2_ prepared by sol–gel method, were prepared by incipient wetness impregnation method with using ruthenium (III) nitrosylnitrate solution (Alfa Aesar) as Ru precursor. The TiO_2_ support was impregnated with the ruthenium (III) nitrosylnitrate solution and then dried at room temperature for 6 h. After that, the resulting catalyst was dried in oven at 110 °C for 12 h and calcined in air at 550 °C for 4 h with heating rate of 10 °C/min. The monometallic 1.5 wt% Co/TiO_2_ catalyst was also prepared by incipient wetness impregnation under similar condition using cobalt naphthenate solution (Sigma Aldrich).

### Preparation of the bimetallic Ru-Co/TiO_2_ catalyst

The bimetallic Ru-Co/TiO_2_ catalysts were prepared by co-incipient wetness impregnation with a nominal Ru loading of 1.5 wt% and Co loadings of 0.2, 0.4, 0.6, and 0.8 wt%. The ruthenium (III) nitrosylnitrate (Alfa Aesar) and cobalt naphthenate (Sigma Aldrich) were used as Ru and Co precursors, respectively. The TiO_2_ anatase support was impregnated with the mixture solution of ruthenium (III) nitrosylnitrate and cobalt naphthenate and then dried at room temperature for 6 h. After that, the resulting catalyst was dried in oven at 110 °C for 12 h and calcined in air at 550 °C for 4 h with heating rate of 10 °C/min.

### Catalyst characterization

The XRD patterns were collected on a Bruker D8 Advance X-ray diffractometer with Ni-filter CuK_α_ radiation from 20° to 80° 2θ and the crystallite size was determined by using the Scherrer’s equation. The physical characteristics of the catalysts, including specific BET surface area, pore volume, and average pore size diameter, were analyzed by using N_2_ physisorption technique on a Micromeritics ASAP 2020 automated system. The TEM micrographs of the catalyst were observed by using a JEOL-JEM 2010 transmission electron microscope using energy-dispersive X-ray detector operated at 200 kV. The XPS was conducted on a Kratos AMICUS X-ray photoelectron spectroscopy with Mg K_α_ X-ray source as a primary excitation and KRATOS VISION II software. The XPS spectra of C1s line at binding energy of 285.0 eV was specified as the internal standard. The metal dispersion of the catalysts was evaluated by CO pulse chemisorption using a Micromeritics ChemiSorb 2750 pulse chemisorption system. The stoichiometry of CO chemisorption was CO/Ru = 1^23^. Prior to chemisorption, the catalyst was reduced under H_2_ (25 cm^3^/min) at 300 °C for 2 h. Afterwards, the sample was maintained at 300 °C under helium flow (25 cm^3^/min) for 0.5 h for removing the physical adsorption of H_2_. Afterwards, the catalyst was cooled down to 30° and then the CO was injected and chemisorbed over the reduced catalyst until the TCD signal due to the effluent CO gas was unchanged. The reducibility of the catalyst was investigated by using H_2_-temperature programmed reduction technique on a Micromeritics ChemiSorb 2750 with ChemiSoft TPx software. Prior to H_2_-TPR, the catalyst was pretreated with N_2_ (25 cm^3^/min) at 200 °C for 1 h and then cooled down to room temperature. After that, the mixture of 10% H_2_ in Ar (25 cm^3^/min) was introduced into the pretreated catalyst. The H_2_-TPR profile was recorded at a ramp rate of 10 °C/min from 30 to 800 °C.

### Catalytic reaction study

To study the catalytic performances of the catalysts, the liquid-phase selective hydrogenation of furfural to FA was carried out under mild reaction conditions (50 °C and 2 MPa H_2_). Prior to the reaction study, the catalyst was reduced under H_2_ flow (25 cm^3^/min) for 2 h at 300 °C. Approximately 0.05 g of reduced catalyst was added into the reactant mixture containing 50 µl of furfural (Sigma Aldrich) and 10 ml of methanol (Sigma Aldrich) in 100 cm^3^ stainless steel autoclave reactor (JASCO, Tokyo, Japan). The selective furfural hydrogenation reaction was carried out at 2 MPa H_2_ pressure and 50 °C for 2 h. The liquid product was analyzed by gas chromatography equipped with a flame ionization detector (FID) and Rtx-5 capillary column.

## Results and discussion

### Monometallic Ru catalysts supported on different TiO_2_ structures

#### Catalyst characterization

**Figure 1:**
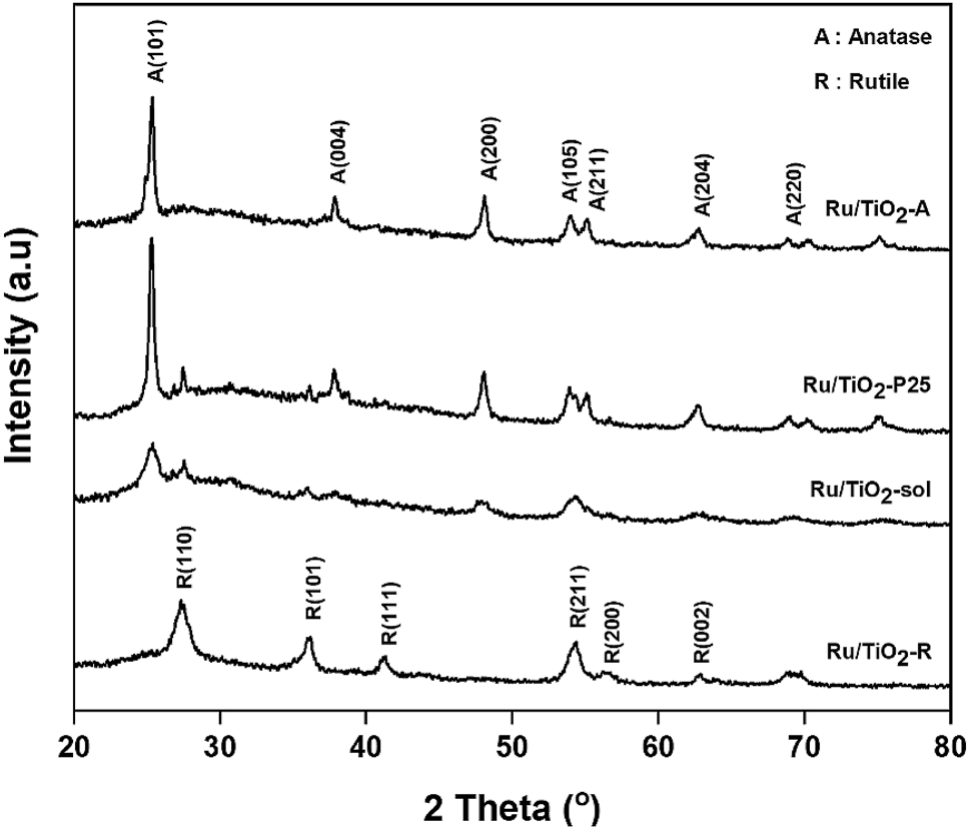
XRD patterns of the monometallic Ru supported on different TiO_2_ structures*.*

The XRD patterns of the monometallic Ru supported on different TiO_2_ structures are shown in Fig. 1. The XRD characteristic peaks showed anatase phase at 2θ = 25° (major), 37°, 48°, 55°, 56°, 62°, 71°, 75° and rutile phase at 27° (major), 36°, 42°, and 57°. The TiO_2_-prepared by sol gel method consisted of both of anatase and rutile phases. No characteristic peaks of Ru species could be detected for all the catalysts because of low content of Ru loading and/or well-dispersion of Ru on TiO_2_ supports^24^. The average TiO_2_ crystallite size and phase composition are given in Table 1. The phase composition of the anatase (W_A_) and rutile (W_R_) phases in the synthesized samples was calculated from the XRD patterns using by the Spurr and Meyer’s Eqs. (1) and (2), as follows^25^:

1 $$ {\text{WR}} = \frac{1}{{1 + 0.8\left( {\frac{{{\text{I}}_{{\text{A}}} }}{{{\text{I}}_{{\text{R}}} }}} \right)}} \times 100 $$

2 $$ {\text{W}}_{{\text{A}}} \, = \,100\, - \,{\text{W}}_{{\text{R}}} $$

**Table 1:** Physical properties and H_2_ consumptions of the monometallic Ru supported on different TiO_2_ structures*.*

Sample	Crystallite size of TiO_2_ (nm)^a^	Phase composition		N_2_ physisorption results			Ru dispersion (%)		H_2_ consumption of Ru-TiO_x_ sites (mmol/g)
		Anatase (%)	Rutile (%)	BET surface area (m^2^/g)	Pore volume (cm^3^/g)	Avg. pore size (nm)	CO Chemisorpsion^c^	TEM^d^	
Ru/TiO_2_-A	27.9	97	3	50	0.32	22.0	24.7	34.9	2.72
Ru/TiO_2_-P25	27.9	87	13	53	0.26	15.5	25.2	36.9	1.94
Ru/TiO_2_-sol	8.6	75	25	106	0.21	5.0	21.8	31.5	2.33
Ru/TiO_2_-R	16.9^b^	5	95	42	0.19	17.9	28.3	40.3	3.12

^a^Crystallite size was calculated from Scherrer’s equation.

^b^Crystallite size of rutile.

^c^Stoichiometry of CO chemisorption was CO : Ru = 1:1.

^d^Estimated assuming clean spherical average particle size.

where I_A_ and I_R_ are the integrated intensity of the (101) reflection of the anatase phase and the (110) reflection of the rutile phase, respectively. For TiO_2_ having major anatase phase, the average TiO_2_ crystallite size was ranged in the order of Ru/TiO_2_-sol (8.6 nm) < Ru/TiO_2_-P25 (27.9 nm) = Ru/TiO_2_-A (27.9 nm) whereas the crystallite size of TiO_2_ rutile phase was ca. 17 nm for Ru/TiO_2_-R catalyst.

The N_2_ physisorption results including BET specific surface area, pore volume, and average pore diameter of all monometallic Ru supported on different TiO_2_ structures are also presented in Table 1. The BET specific surface area, pore volume, and average pore diameter of Ru/TiO_2_-A and Ru/TiO_2_-P25 were not much different. In addition, the Ru/TiO_2_-sol showed higher surface area but smaller average pore diameter as compared to those supported on TiO_2_ having major anatase phase. High surface area of Ru/TiO_2_-sol was attributed to the heat treatment only at 350 °C upon sol–gel technique, which led to decrease in aggregation of TiO_2_ particles and smaller TiO_2_ crystallite size^26^. The Ru/TiO_2_-R catalyst showed the lowest surface area ca. 42 m^2^/g with the pore volume 0.19 cm^3^/g, respectively.

**Figure 2:**
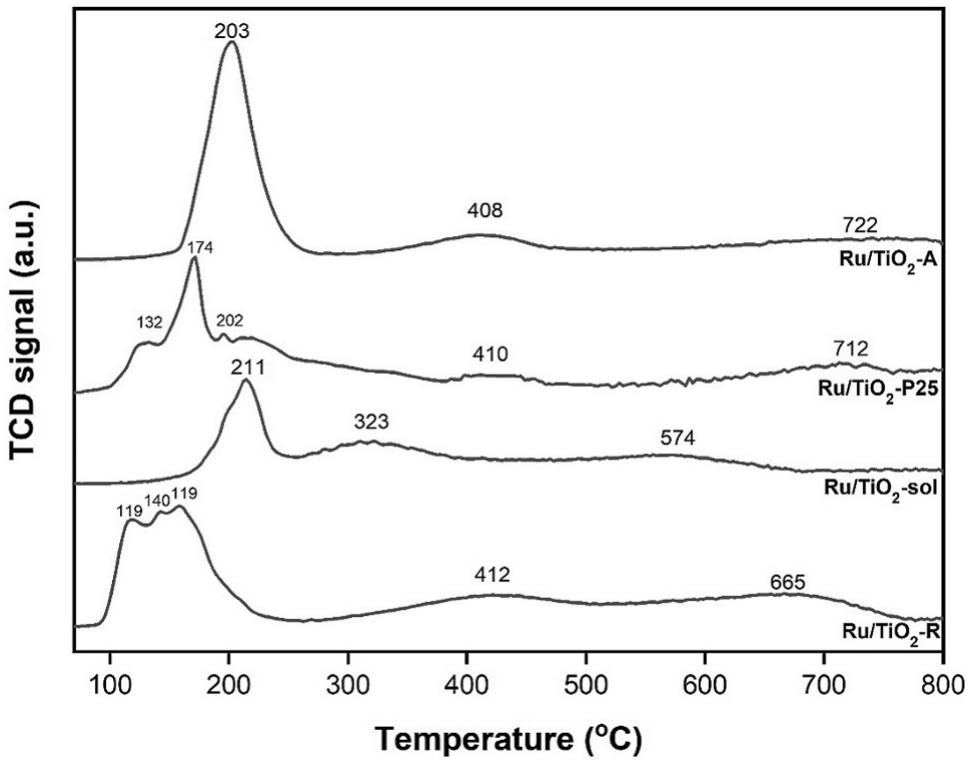
H_2_-TPR profiles of the monometallic Ru supported on different TiO_2_ structures.

The H_2_-TPR technique was used to study the interaction between metal and support and the reducibility of the Ru-based catalysts supported on different TiO_2_ structures and the H_2_-TPR profiles are shown in Fig. 2. All the Ru-based catalysts showed three main reduction peaks consisting of the first reduction peak at 100–250 °C corresponding to the reduction of Ru oxides to Ru^0^ metal^27–29^, the reduction peak in the range of 300–450 °C corresponding to Ru interacting with TiO_2_ support in the form of Ru-TiO_x_ sites^27–30^, and the broad reduction peak at 570–730 °C assigning to the reduction of surface capping oxygen of TiO_2_ support (partial reduction of TiO_2_). Considering the reduction peak of Ru oxides to Ru^0^ metal, a series of multiple reducible peaks of the Ru oxides reduction were observed on the Ru/TiO_2_-P25 and Ru/TiO_2_-R catalysts probably due to the presence of different Ru ion species and/or different Ru particle sizes in different environments on the surface of the support^23,30,31^. The crystallization of Ru particles was reported to be responsible for the reduction peak of Ru oxides. The reduction peak at lower temperature was attributed to the reduction of well-dispersed RuO_x_ particles, while the reduction peak at higher temperature corresponded to the reduction of RuO_x_ with larger particle size^23,24,32^. On the other hand, the Ru/TiO_2_-A and Ru/TiO_2_-sol showed only single sharp reduction peak, suggesting to the homogeneity in particle size distribution of Ru species. Thus, the particle size of Ru based on the reduction peak of Ru oxides could be ranged in the order of Ru/TiO_2_-R < Ru/TiO_2_-P25 < Ru/TiO_2_-A < Ru/TiO_2_-sol. According to the reduction peak due to the Ru interacting with TiO_2_ support in the form of Ru-TiO_x_ sites, the reduction peaks were not significantly different for the Ru/TiO_2_-R, Ru/TiO_2_-P25, and Ru/TiO_2_-A catalysts. In addition, the reduction peak due to the Ru interacting with TiO_2_ support for the Ru/TiO_2_-sol was found to appear at lower temperature (323 °C) as compared to the other catalysts, indicating to weaker interaction between Ru and TiO_2_-prepared by sol-gel^28^. Moreover, the partial reduction of TiO_2_-prepared by sol–gel was found to easily occur as shown by the lower reduction temperature at 574 °C which could be explained that its thermodynamic and structure were less stable than the other TiO_2_ supports. The amount of H_2_ consumption for the reduction of Ru interacting with TiO_2_ support in the form of Ru-TiO_x_ sites is given in Table 1 and the results were found to be in the order of Ru/TiO_2_-R > Ru/TiO_2_-A > Ru/TiO_2_-sol > Ru/TiO_2_-P25.

The Ru dispersion on the Ru-based catalysts supported on different TiO_2_ structures was determined by using CO pulse chemisorption based on the stoichiometry of CO/Ru = 1^23^ and the results are shown in Table 1. The shape of Ru particles was assumed to be cubic with five sides exposed to the gas reactant^23,32^. High dispersion of Ru was found on both the Ru/TiO_2_-R and Ru/TiO_2_-P25 catalysts, which corresponded to the shift of the reduction peak of well-dispersed RuO_x_ towards lower temperature. In addition, the Ru dispersions of the Ru-TiO_2_-sol and Ru/TiO_2_-A were lower than that of Ru/TiO_2_-R and Ru/TiO_2_-P25, indicating to larger Ru particle size. The characteristics of 5 wt.% Ru catalysts supported on different TiO_2_ supports (anatase, rutile, and P25) were studied and reported that the Ru dispersion increased monotonically from anatase to P25 to rutile while the trend of Ru particle size was decreased^30^. Although the sintering of Ru particles might occur upon high temperature reduction step, the lattice matching of RuO_x_ and TiO_2_ rutile during calcination as well as a stronger interaction between metal and oxygen vacancies sites during reduction would favor the stabilization of small Ru particles on the TiO_2_ rutile surface, thus leading to the enhancement of Ru dispersion on rutile^30^. Comparing to the Ru/TiO_2_-P25, the Ru dispersion of Ru/TiO_2_-sol was lower despite higher rutile phase composition since the thermodynamic and structure of TiO_2_-prepared by sol–gel were less stable, thus leading to weak interaction between Ru and oxygen vacancies sites in TiO_2_. We also estimated Ru dispersion based on the TEM images, which indicated much higher Ru dispersion values than the CO chemisorption analysis. These results suggested that some Ru particles were covered by the support. However, both CO chemisorption and TEM analyses yielded the same order of Ru dispersion: Ru/TiO2-R > Ru/TiO2-P25 > Ru/TiO2-A > Ru/TiO2-Sol catalysts. These results are in agreement with the previous studies^33–36^. We considered that Ru dispersion was affected by several factors, such as isoelectric point, nucleation and growth of Ru, ionic strength between Ru and support, etc.

**Figure 3:**
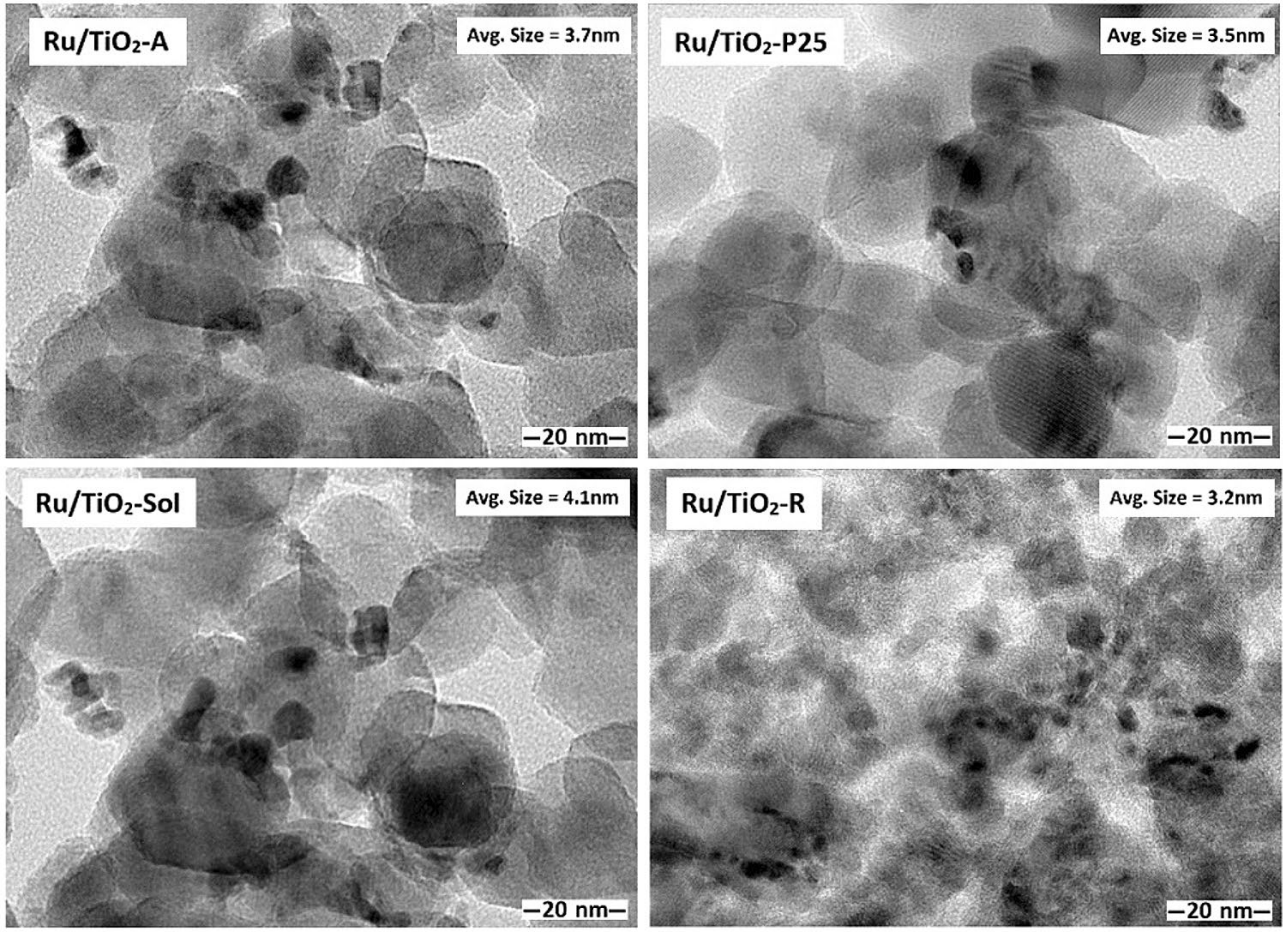
TEM images of the monometallic Ru based catalysts supported on different TiO_2_ structures.

The TEM images of the monometallic Ru based catalysts supported on different TiO_2_ structures are shown in Fig. 3. The average metal particle size of Ru/TiO_2_-A, Ru/TiO_2_-P25, Ru/TiO_2_-Sol and Ru/TiO_2_-R were 3.7, 3.5, 4.1 and 3.2 nm, respectively. The results were correlated well with the particle size of Ru based on the reduction peak of Ru oxides revealed by H_2_-TPR.

**Figure 4:**
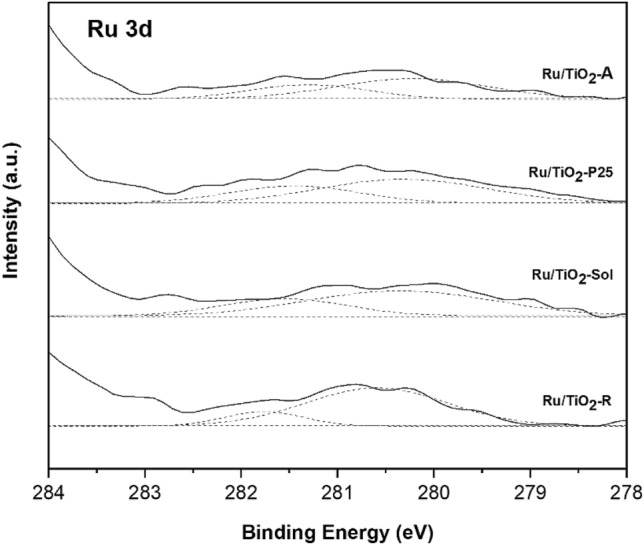
XPS Ru 3*d* core level spectra of the monometallic Ru based catalysts supported on different TiO_2_ structures.

The binding energy of Ru 3*d*_5/2_ for monometallic Ru based catalysts supported on different TiO_2_ structures are illustrated in Fig. 4. Typical binding energy of metallic Ru^0^ was reported to be 279.75 ± 0.37 eV. According to our study, the binding energy of Ru3*d*_5/2_ for the monometallic Ru/TiO_2_ was observed at 280.2 and 281.5 + 0.2 eV which are assigned to metallic Ru and RuO_2_, respectively^37,38^. After the deconvolution of the corresponding spectra, the percentages of the RuO_2_ species in Ru/TiO_2_-A, Ru/TiO_2_-P25, Ru/TiO_2_-Sol and Ru/TiO_2_-R catalysts were 36.4, 35.1, 31.0 and 17.5%, respectively. The percentages of RuO_2_ species of Ru/TiO_2_-P25, Ru/TiO_2_-Sol and Ru/TiO_2_-R were lower than Ru/TiO_2_-A catalyst, resulting in lower catalytic performance. According to previous studies^39^ indicated that a large fraction of RuO_2_ species of Ru/C catalysts improve the catalytic performance for lactic acid and butanone hydrogenation. These results suggest that the higher fraction of RuO_2_ species is the catalytically active species, which is advantageous to the for selective hydrogenation of furfural to FA.

#### Catalytic study in liquid-phase selective hydrogenation of furfural

The catalytic performances of Ru based catalysts supported on different TiO_2_ structures were studied in the liquid-phase selective hydrogenation of furfural to FA under mild conditions. According to our study, the products formed upon furfural hydrogenation were FA as the desired product and 2-furaldehyde dimethyl acetal as a by-product, which is formed by acetalization of furfural in alcoholic solvents such as methanol and ethanol^40^. The catalytic performances of Ru based catalysts supported on different TiO_2_ structures are shown in Table 2. The catalytic activity was found to be ranged in the order: Ru/TiO_2_-A > Ru/TiO_2_-P25 > Ru/TiO_2_-sol > Ru/TiO_2_-R which corresponded to the anatase phase composition in the TiO_2_ supports. Despite its lower Ru dispersion, the Ru/TiO_2_-A catalyst exhibited superior catalytic activity due to high anatase phase composition. It is suggested that the anatase phase composition in the TiO_2_ support played a main role in the catalytic activity of Ru-based catalysts supported on TiO_2_ for furfural hydrogenation. According to the literature, the subsurface oxygen vacancies in the reduced catalysts supported on TiO_2_ anatase have been reported to be favorable adsorption sites for hydrogen atoms^41^. In other words, the molecular hydrogen could not interact strongly with the rutile TiO_2_-supported catalysts which diminished the catalytic activity in hydrogenation reaction^42^. In addition, the selectivity to FA was related to the interaction between Ru and TiO_2_ support in the form of Ru-TiO_x_ sites. A close contact between Ru active metal and partially reduced species of TiO_2_ support resulted in the surface decoration as the geometric effect and such presence of Ru-TiO_x_ interface sites would create the polarity which favored the interaction of the lone pair electron of oxygen atom of the C=O bond in furfural^28,43^. The Ru/TiO_2_-sol showed relatively low selectivity to FA due to weak interaction between Ru and TiO_2_ as observed in the shift of Ru-TiO_x_ reduction peak toward lower temperature. Considering the Ru/TiO_2_-R, Ru/TiO_2_-A, and Ru/TiO_2_-P25 catalysts, the interaction between Ru and TiO_2_ support was not much different as observed from the position of Ru-TiO_x_ reduction peak in H_2_-TPR profiles. The selectivity to FA for the Ru/TiO_2_-R, Ru/TiO_2_-A, and Ru/TiO_2_-P25 catalysts were found to increase with the amount of H_2_ consumption for the reduction of Ru interacting with TiO_2_ support in the form of Ru-TiO_x_ sites as given in Table 1. Although the greater amount of Ru-TiO_x_ sites was observed on the Ru/TiO_2_-R, the catalytic performance was very low due to relative low catalytic activity in furfural hydrogenation. Among the Ru-based catalysts supported on different TiO_2_ structures studied, the Ru/TiO_2_-A is the most promising catalyst for further modification by Co addition as a second metal.

**Table 2:** Catalytic performances of the monometallic Ru supported on different TiO_2_ structures in liquid-phase selective hydrogenation of furfural to FA.

Catalysts	Furfural conversion (%)	Selectivity to FA (%)	Selectivity to solvent product (%)
Ru/TiO_2_-A	31.8	90.0	10.0
Ru/TiO_2_-P25	20.0	85.3	14.7
Ru/TiO_2_-Sol	17.5	75.1	24.9
Ru/TiO_2_-R	4.4	96.3	5.7

Reaction conditions: 50µL of furfural in 10 ml of methanol under 50 °C and 2 MPa H_2_ with a 50 mg of catalyst for 120 min.

FA as a desired product and 2-furaldehyde dimethyl acetal as a solvent product.

### Bimetallic Ru-Co catalysts supported on TiO_2_ anatase support

#### Catalyst characterization

According to previous part, the TiO_2_ anatase (TiO_2_-A) was chosen to use as the support for further modification of the Ru-based catalysts. The bimetallic Ru-Co catalysts supported on TiO_2_ anatase were prepared by co-incipient wetness impregnation in order to study the modification by Co addition as a second metal for selective hydrogenation of furfural to FA. The BET specific surface area, pore volume, and average pore diameter of all the bimetallic Ru-Co catalysts as presented in Table 3 were not much different, suggesting that the Co addition with 0.2–0.8 wt% Co loadings did not affect the physical properties of the Ru-based catalysts. In addition, the average TiO_2_ crystallite sizes were found to be in the range of 28–32 nm and the anatase phase composition after Co addition was similar to the monometallic Ru/TiO_2_ catalyst.

**Table 3:** Physical properties results of the monometallic Ru and bimetallic Ru-Co catalysts supported on TiO_2_ anatase*.*

Sample	Crystallite size of TiO_2_ (nm)^a^	Phase composition		N_2_ physisorption results			Ru dispersion (%)	
		Anatase (%)	Rutile (%)	BET surface area (m^2^/g)	Pore volume (cm^3^/g)	Avg. Pore size (nm)	CO Chemisorpsion^b^	TEM^c^
Ru/TiO_2_	27.9	97	3	50	0.32	22.0	24.7	34.9
Ru-0.2Co/TiO_2_	27.9	95	5	42	0.36	29.2	19.7	28.7
Ru-0.4Co/TiO_2_	30.7	95	5	42	0.30	23.8	15.2	24.8
Ru-0.6Co/TiO_2_	30.7	96	4	45	0.31	22.3	13.9	22.6
Ru-0.8Co/TiO_2_	32.4	93	7	41	0.32	25.9	10.8	20.8

^a^ Crystallite size was calculated from Scherrer’s equation.

^b^ Stoichiometry of CO chemisorption was CO : Ru = 1:1.

^c^ Estimated assuming clean spherical average particle size.

**Figure 5:**
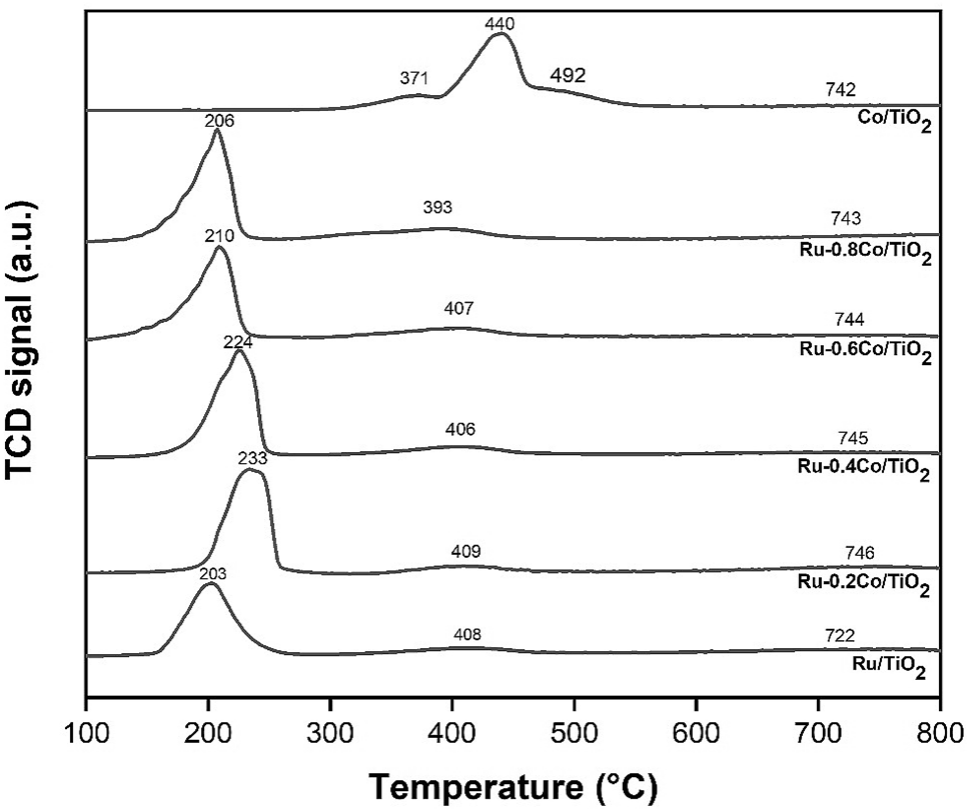
H_2_-TPR profiles of the monometallic Ru and bimetallic Ru-Co catalysts supported on TiO_2_ anatase.

The interaction between Ru-Co and between metals-TiO_2_ and the reduction behavior of monometallic Ru and Co and bimetallic Ru-Co catalysts were studied by using H_2_-TPR technique and the H_2_-TPR profiles are shown in Fig. 5. The monometallic Co/TiO_2_ showed the main peak at 440 °C with the shoulder peaks at 371 °C and 492 °C. The reduction of Co_3_O_4_ supported on TiO_2_ consisted of two stages: Co_3_O_4_ → CoO → Co^0^ metal^44–47^. The shoulder peak at lower temperature (371 °C) was related to the primary reduction of Co_3_O_4_ to CoO, while the subsequent reduction of CoO to Co^0^ metal mainly appeared at higher temperature (440 °C). In addition, the shoulder peak at higher temperature (492 °C) could be attributed to the reduction of Co species strongly interacting with TiO_2_ support^45–47^. The broad peak at 742 °C was assigned to the reduction of surface capping oxygen of TiO_2_ support (partial reduction of TiO_2_). The monometallic Ru/TiO_2_ exhibited three reduction peaks consisting of the reduction of Ru oxides to Ru^0^ metal (at 203 °C)^27–29^, the reduction of Ru species interacting with TiO_2_ support as the Ru-TiO_x_ sites (at 408 °C)^27–30^, and the partial reduction of TiO_2_ support (at above 700 °C), respectively.

Considering the bimetallic Ru-Co catalysts, a slight shift in the reduction peak of Ru oxides to Ru^0^ metal towards higher temperature could be observed as compared to the monometallic Ru/TiO_2_. Such results suggest an interaction between Ru and Co, which were closely interacting, probably in the form of bimetallic RuCo alloy^28,48^. Similar trend has previously been found on the bimetallic Ru-Sn catalysts^28^. Moreover, this shift to higher temperature could also be attributed to the decrease in Ru dispersion in the presence of Co as a second metal. The Co addition with 0.2 wt% showed the largest shift in the Ru reduction peak towards higher temperature (233 °C). Nevertheless, the Ru reduction peak was reversely shifted to lower temperature with increasing Co addition up to 0.8 wt%, probably due to the formation of Ru-Co alloying system. According to the electronegativity, a direction of net charge transfer would occur from Co to Ru in Ru-Co alloy, thus promoting the reducibility of Ru oxides. The reduction peaks due to Co oxides did not appear in the H_2_-TPR profiles of all bimetallic Ru-Co catalysts. Taking into account that the main reduction peak of Co oxides in the monometallic Co/TiO_2_ occurred at 440 °C, the disappearance of reduction peak due to Co oxides would be indicating a strong interaction between Ru and Co. The Ru addition in the bimetallic Ru-Co catalysts was reported to lower the reduction temperature of Co oxides^49,50^. This phenomenon could be explained that the Ru oxides would be firstly reduced to Ru metals at rather lower temperature and then the dissociative adsorption of H_2_ on the Ru metals caused the H spillover to Co oxides resulting in the reduction of Co oxides to Co metals. In addition, the broad reduction peak of metals-TiO_x_ sites was found to be not significantly different in the range of 400–410 °C for all Ru-based catalysts except the Ru-0.8Co/TiO_2_, in which the reduction peak shifted to lower temperature at 393 °C, suggesting to weaker interaction between Ru and TiO_2_ probably due to excess Co loading (0.8 wt%).

**Figure 6:**
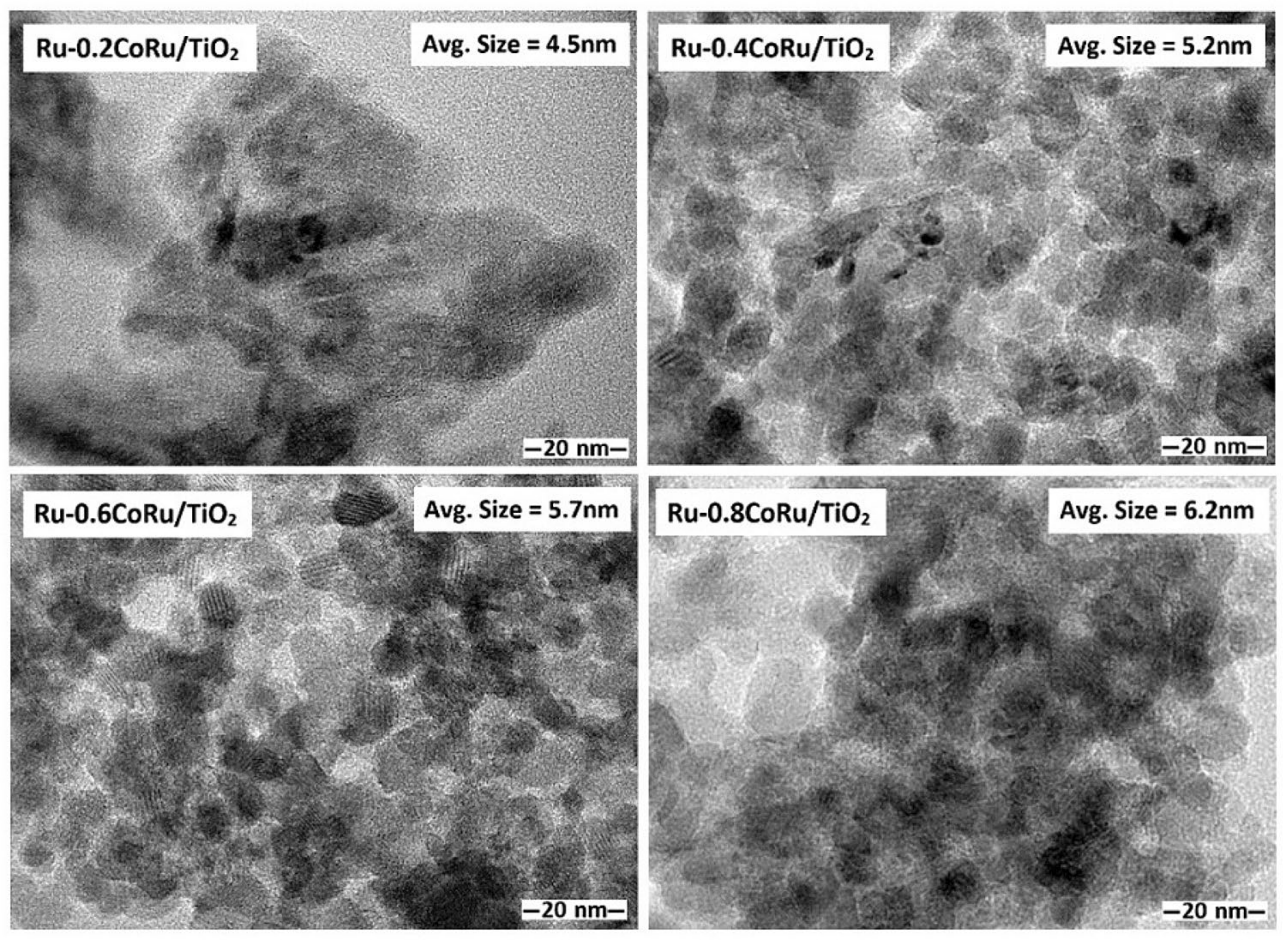
TEM images of the bimetallic Ru-Co catalysts supported on TiO_2_ anatase.

**Figure 7:**
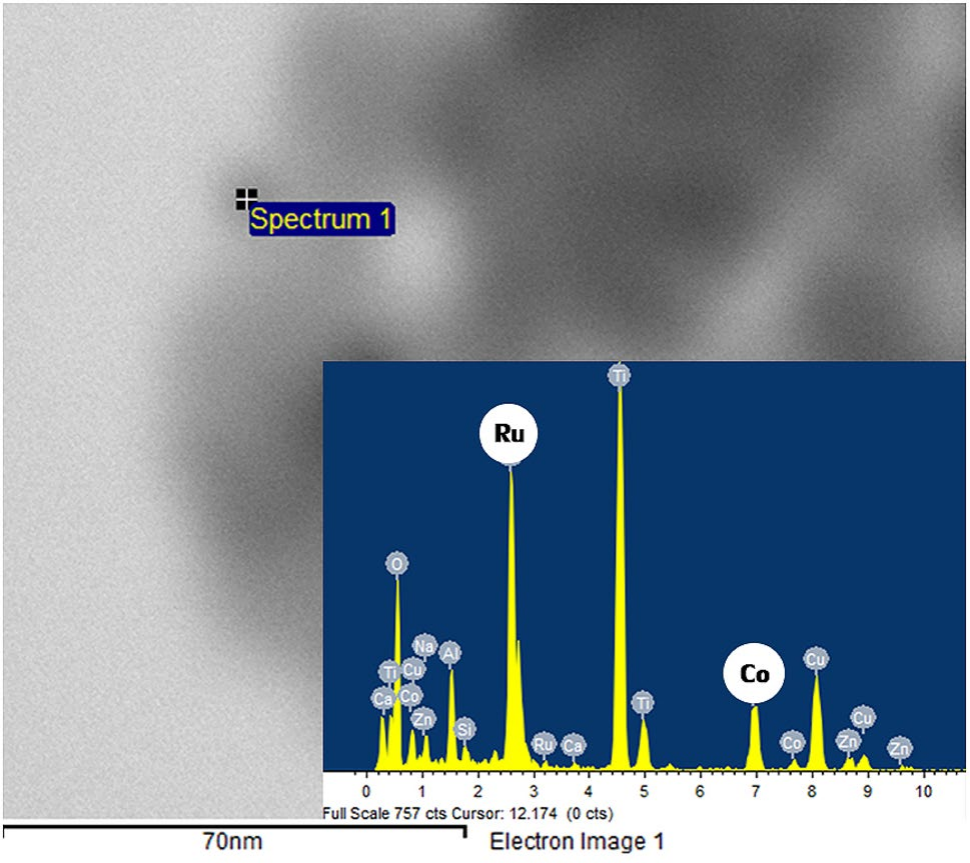
EDX spectra of the bimetallic Ru-0.6Co/TiO_2_ catalyst.

**Figure 8:**
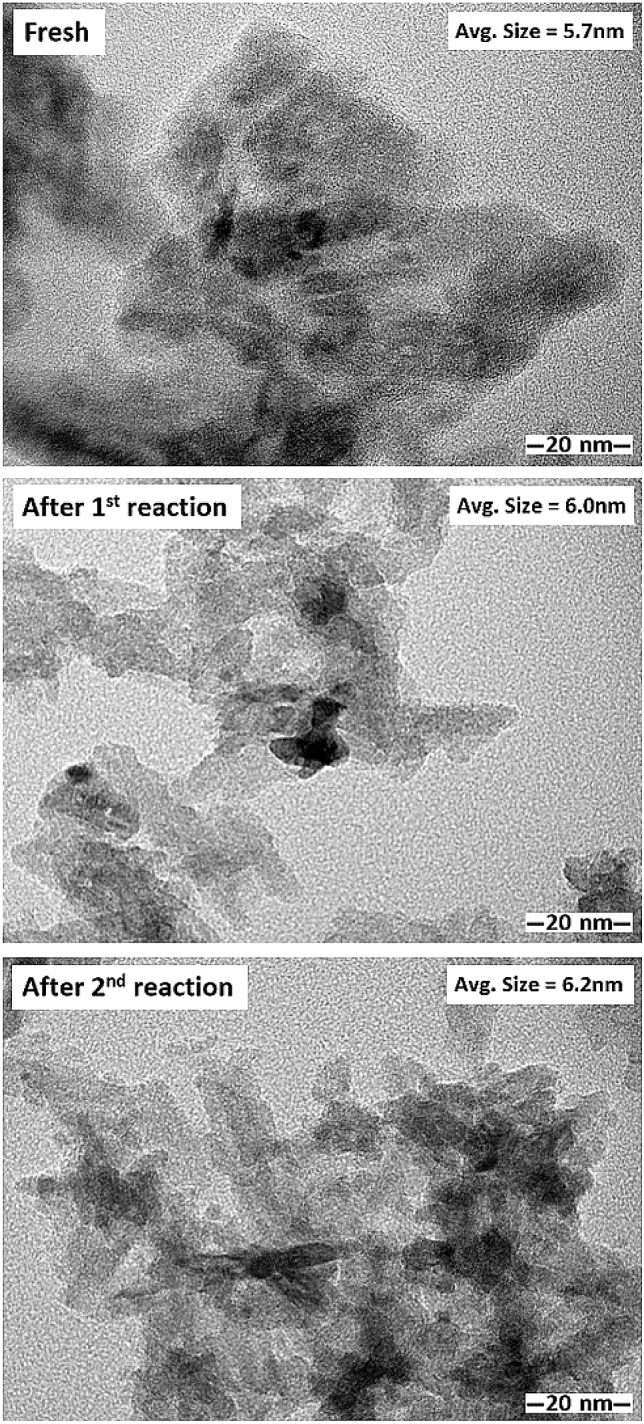
TEM images of the used Ru-0.6Co/TiO_2_ catalyst after 1st and 2nd reactions.

The TEM images of the monometallic Ru/TiO_2_ and bimetallic Ru-Co/TiO_2_ catalysts with different Co loadings are shown in Fig. 6. For the monometallic Ru/TiO_2_ catalyst, the Ru particles appeared as small dark spots with an average particle size of ca. 3.7 nm and it seemed to be uniformly dispersed on the TiO_2_ support. In addition, the average particle size of dark spots for the bimetallic Ru-Co/TiO_2_ was found to increase with increasing Co addition. The TEM–EDX analysis confirmed the presence of both Ru and Co as appeared in the small dark spots observed in the TEM images (Fig. 7) of bimetallic Ru-Co catalysts, indicating the formation of bimetallic Ru-Co nanoparticles. The average particle size of bimetallic Ru-Co nanoparticles was ca. 4.5, 5.2, 5.7, and 6.2 nm for bimetallic Ru-0.2Co/TiO_2_, Ru-0.4Co/TiO_2_, Ru-0.6Co/TiO_2_, and Ru-0.8Co/TiO_2_ catalysts, respectively. An increase in particle size due to the second metal addition has also been found in the modification of Ru-based catalysts by Sn addition and the increase in particle size of bimetallic Ru-Sn/C catalysts was attributed in part to the selective deposit of Sn on Ru^8^. The presence of both Ru and Co in the bimetallic nanoparticles as small dark spots in TEM–EDX results was consistent with the close interaction between Ru and Co as observed in the H_2_-TPR results. The TEM images of used Ru-0.6Co/TiO_2_ catalyst after 1st and 2nd reactions are presented in Fig. 8. The average metal particle size of fresh and used Ru-0.6Co/TiO2 catalyst after 1st and 2nd reaction were 5.7, 6.0 and 6.2 nm, respectively. The average metal particle size increased after the 2nd successive use as compared to fresh Ru-0.6Co/TiO_2_ catalyst. This can be attributed to the metal particle sintering which may cause the decrease in catalytic performance.

**Figure 9:**
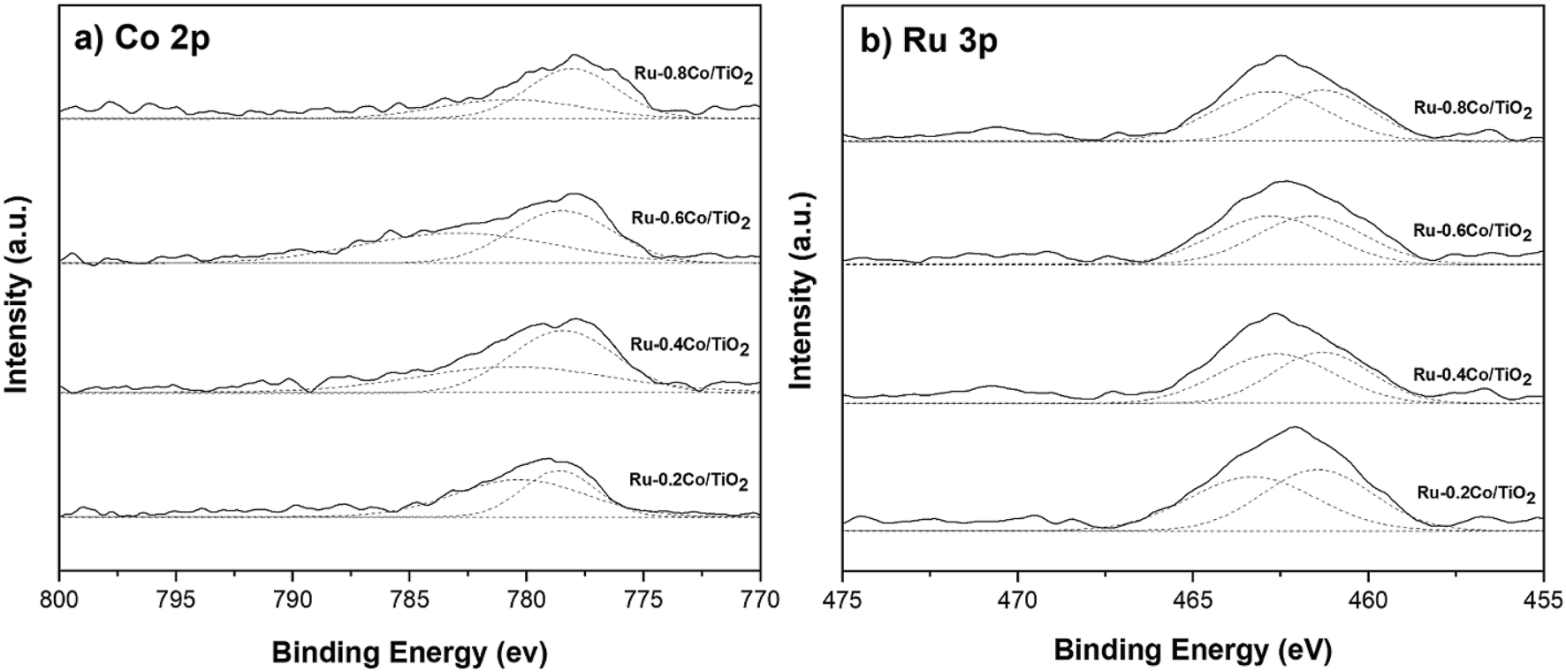
XPS Co 2*p* and Ru 3*p* core levels spectra of the bimetallic Ru-Co catalysts supported on TiO_2_ anatase.

**Figure 10:**
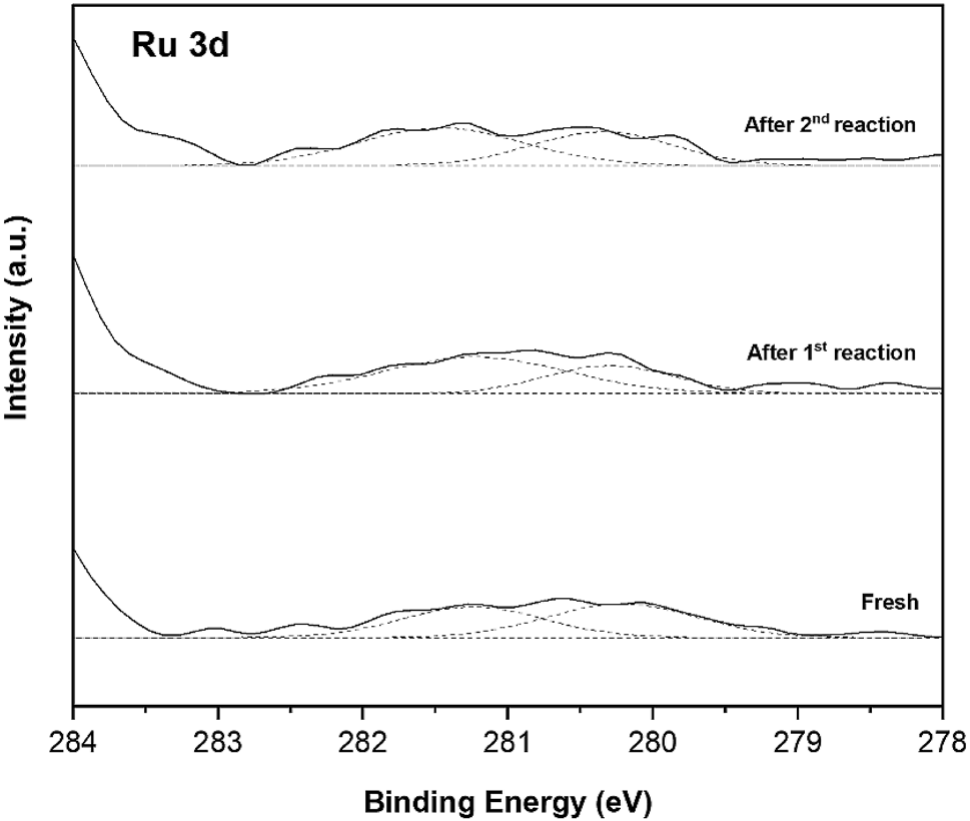
XPS Ru 3*d* core levels spectra of used Ru-0.6Co/TiO_2_ catalyst after 1st and 2nd reactions.

XPS analysis of bimetallic RuCo catalysts are presented in Fig. 9. The Co 2*p*_3/2_ spectra (Fig. 9a) exhibit two predominant peaks located at 778.6 and 780.9 eV, demonstrating the formation of metallic Co and Co^2+^ species, respectively. The peaks of Ru 3*p*_3/2_ spectra (Fig. 9b) at 461.2 and 463.4–463.0 eV can be attributed to metallic Ru and Ru oxides species, respectively. With increasing Co addition, the XPS peaks of Ru 3*p* shifted towards lower binding energies while the Co 2*p* peaks shifted to higher binding energy, indicating the charge transfer between Ru and Co. The binding energy shifts of the Co 2*p* and Ru 3*p* are in the opposite direction, which could result from the charge transfer from less electronegative Co to more electronegative Ru^51,52^. Thus, the effective electron transfer between Co and Ru in the RuCo alloy in bimetallic catalysts can give rise to improve the catalytic performance. Figure 10 shows the Ru 3*d* core-level spectra for used Ru-0.6Co/TiO_2_ catalyst after 1st and 2nd reactions. There is a clear shift of RuO_2_ in the binding energies of 281.1 to 281.5 eV, which is largely attributed to the different levels of hydration and consistent with bulk hydrated and anhydrous ruthenium oxides^53–55^. The higher shifts in energy may be caused by an increase in the mean particle size revealed by TEM.

The Ru dispersion on monometallic Ru/TiO_2_ and bimetallic Ru-Co/TiO_2_ catalysts with different Co loadings were determined by using CO pulse chemisorption based on the stoichiometry of CO/Ru = 1^23^ and the results are shown in Table 3. The addition of Co decreased the amount of CO chemisorption and consequently lowered the Ru dispersion on the bimetallic Ru-Co catalysts. As a result of lower Ru dispersion, the larger metal particle sizes were calculated with increasing Co addition for the bimetallic Ru-Co catalysts, suggesting to the sintering. According to the TEM–EDX results, the particle size of bimetallic Ru-Co nanoparticles was found to increase with increasing Co addition while the Ru dispersion decreased. The Ru dispersion seemed to depend on the particle size of bimetallic Ru-Co nanoparticles in which the agglomeration of bimetallic nanoparticles at high loading led to lower Ru dispersion^31,56^. Nevertheless, no distinct XRD peaks of Ru and Co could be detected in XRD results of the bimetallic Ru-Co catalysts. Thus, lower CO uptake observed on the bimetallic Ru-Co catalysts might not be due to the metal sintering. The decrease in CO uptake was rather be due to the change in CO adsorption stoichiometry and/or the alloy formation^57^.

#### Catalytic study in liquid-phase selective hydrogenation of furfural

The catalytic performances of the monometallic Ru/TiO_2_ and bimetallic Ru-Co/TiO_2_ catalysts with different Co loadings are presented in Table 4. The catalytic activity of the bimetallic Ru-Co catalysts was found to be significantly improved with increasing Co addition. In addition, the slight enhancement of selectivity to FA was observed on the bimetallic Ru-Co catalysts. Superior catalytic performances of bimetallic Ru-Co catalysts as compared to the monometallic Ru/TiO_2_ have been attributed to the formation of Ru-Co alloy system. Moreover, the catalytic performance of the monometallic 1.5 wt% Co/TiO_2_ catalyst was also tested and was found to be non-active for furfural hydrogenation. According to Brewer resonance bond-valence theory^58^, the combination of hyper d-electronic character of Ru (being good catalyst as individual metal) and hyper d-electronic character of Co (being poor catalyst as individual metal) forming the alloy system pronounced the synergistic effect, resulting in the improvement of catalytic performances upon Ru-Co alloying. Such effect of the bimetallic Ru-Co alloy has also been reported in catalyzing the hydrogen evolution^59^. With increasing Co addition, the strong interaction between Ru and Co in the Ru-Co alloy system was promoted as revealed by the XPS results. Thus, the catalytic performances of the bimetallic Ru-Co catalysts in the selective hydrogenation of furfural to FA depended not only on the formation of Ru-Co alloying but also the interaction between Ru and Co. Furthermore, the role of Ru which enhanced the reducibility of Co oxides (as shown in the disappearance of reduction peak due to Co oxides in H_2_-TPR results) is another factor for the improved its hydrogenation activity. However, the catalytic activity for the Ru–0.8Co/TiO_2_ catalyst was slightly decreased due probably to weak interaction of metals-TiO_x_ sites as observed in the H_2_-TPR results, which suppressed the C=O activation. Among the catalysts synthesized in this work, the bimetallic Ru-0.6Co/TiO_2_ catalyst was the most efficient catalyst for selective hydrogenation of furfural to FA with 89.4% yield of FA at 2 h reaction time under the reaction conditions used. The Ru–0.6Co/TiO_2_ catalyst was also tested for successive reuse cycles and the results are shown in Table 5. It can be seen that the furfural conversion of Ru-0.6Co/TiO_2_ catalyst decreased during the 2nd successive use to 78.2% while the FA selectivity slightly decreased to about 94%. It can be explained by the large amount of ruthenium oxides and an increase in the mean particle size revealed by XPS and TEM, respectively.

**Table 4:** Catalytic performances of the monometallic Ru and bimetallic Ru-Co catalysts supported on TiO_2_ anatase in liquid-phase selective hydrogenation of furfural to FA.

Catalysts	Furfural conversion (%)	Selectivity to FA (%)	Selectivity to solvent product (%)
Ru/TiO_2_	31.8	90.0	10.0
Ru-0.2Co/TiO_2_	56.9	95.7	4.3
Ru-0.4Co/TiO_2_	81.2	95.7	4.3
Ru-0.6Co/TiO_2_	91.7	97.5	2.5
Ru-0.8Co/TiO_2_	86.4	96.1	3.9

Reaction conditions: 50µL of furfural in 10 ml of methanol under 50 °C and 2 MPa H_2_ with a 50 mg of catalyst for 120 min.

FA as a desired product and 2-furaldehyde dimethyl acetal as a solvent product.

**Table 5:** Catalytic performances of the Ru-0.6Co/TiO_2_ catalysts on each successive reuse cycle in liquid-phase selective hydrogenation of furfural to FA.

Cycle no	Furfural conversion (%)	Selectivity to FA (%)	Selectivity to solvent product (%)
1st	91.7	97.5	2.5
2nd	82.5	95.2	4.8
3rd	78.2	93.8	6.2

Reaction conditions: 50µL of furfural in 10 ml of methanol under 50 °C and 2 MPa H_2_ with a 50 mg of catalyst for 120 min.

FA as a desired product and 2-furaldehyde dimethyl acetal as a solvent product.

## Conclusions

The Ru-based catalysts supported on TiO_2_ for liquid-phase selective hydrogenation of furfural to FA under mild conditions (50 °C and 2 MPa H_2_) were studied and modified in terms of TiO_2_ structure and the Co addition as a second metal. The TiO_2_ anatase was suitable to use as a support for Ru-based catalysts as the high anatase phase TiO_2_ was the favorable adsorption sites for hydrogen. In addition, the strong interaction between Ru and TiO_2_ in the form of Ru-TiO_x_ sites as shown by the H_2_-TPR results promoted the selectivity to FA. Considering the modification by Co addition with different Co loadings, the catalytic performances in terms of furfural conversion and selectivity to FA of the bimetallic Ru-Co catalysts were significantly increased with increasing Co loading. This improvement was attributed to the synergistic effect of Ru-Co alloying system together with the interaction between Ru and Co as observed in the XPS, H_2_-TPR, and TEM–EDX results. With increasing Co addition, the strong interaction between Ru and Co in the Ru-Co alloy system was promoted. The role of Ru in the Ru-Co alloy would increase the reducibility of Co oxides which also accounted in improving the furfural hydrogenation activity. The bimetallic Ru-0.6Co/TiO_2_ catalyst showed the best catalytic performances for selective hydrogenation of furfural to FA with 89.4% yield of FA. Nevertheless, Further addition of Co beyond 0.6 wt% led to a weak interaction of metals-TiO_x_ sites which suppressed the C=O activation, thus lowering the catalytic performance of selective furfural hydrogenation as seen for the Ru–0.8Co/TiO_2_ catalyst.
